# Supplementary material for: Two-way text message interventions and healthcare outcomes in Africa: Systematic review of randomized trials with meta-analyses on appointment attendance and medicine adherence
Source: PLoS One. 2022 Apr 14;17(4):e0266717. doi: 10.1371/journal.pone.0266717 (PMC9009629; doi:10.1371/journal.pone.0266717)
Supplement: S2 File — (PDF) [file pone.0266717.s002.pdf]

S2 File: Literature search strings

Medline – search string

#1	#1<br>short message service*/ OR sms/ OR sms message/ OR Two way ADJ3 SMS/ OR sms messaging/ OR sms reminder*/ OR sms-reminder/ OR text message*/ OR text messaging/ OR text message reminder*/ OR mobile ADJ2 health/ OR mhealth/ OR mhealth ADJ2 intervention/ OR mhealth ADJ2 interventions/ OR mobile phone intervention*/ OR mobile phone call*/ OR cell phone intervention*/ OR cell phone call*/ OR cell phone reminder*/ OR cell phone message*/ OR exp text messaging/ OR texting/ OR textings/ OR exp telemedicine/
#2	Africa South of the Sahara/ OR Africa, Eastern/ OR Africa, Northern/ OR Africa, Southern/ OR Africa, Western/ OR Africa/ OR African/ OR Algeria*/ OR Angola*/ OR Benin*/ OR Botswana/ OR Botswanese/ OR Burkina Faso/ OR Burkinabe/ OR Burundi*/ OR Cameroon*/ OR Cape Verde/ OR Cape Verdean/ OR Central African Republic/ OR Central African/ OR Chad*/ OR Comoros/ OR Comoran/ OR Congo*/ OR Cote dIvoire/ OR Ivorian/ OR Democratic Republic of the Congo/ OR Djibouti*/ OR Egypt*/ OR Equatorial Guinea*/ OR Eritrea*/ OR Ethiopia*/ OR Gabon*/ OR Gambia*/ OR Ghana*/ OR Guinea-Bissau/ OR Bisseau-Guinean/ OR Guinea*/ OR Kenya*/ OR Lesotho/ OR Basotho/ OR Mosotho/ OR Liberia*/ OR Libya*/ OR Madagascar/ OR Madagascan/ OR Mayonette*/ OR Malawi*/ OR Mali*/ OR Mauritania*/ OR Morocco/ OR Moroccan/ OR Mozambique/ OR Mozambicans/ OR Namibia*/ OR Niger*/ OR Nigeria*/ OR Rwanda*/ OR Senegal/ OR Senegalese/ OR Sierra Leone*/ OR Somalia*/ OR South Africa*/ OR South Sudan*/ OR Sudan*/ OR Swaziland/ OR Swazis/ OR Tanzania*/ OR Togo*/ OR Tunisia*/ OR Uganda*/ OR Zambia*/ OR Zimbabwe*/
#3	Exp Africa South of the Sahara/ OR exp Africa, Central/ OR exp Africa, Eastern/ OR exp Africa, Northern/ OR exp Africa, Southern/ OR exp Africa, Western/ OR exp Algeria/ OR exp Angola/ OR exp Benin/ OR exp Botswana/ OR exp Burkina Faso/ OR exp Burundi/ OR exp Cameroon/ OR exp Cape Verde/ OR exp Central African Republic/ OR exp Chad/ OR exp Congo/ OR exp Cote dIvoire/ OR exp Democratic Republic of the Congo/ OR exp Djibouti/ OR exp Egypt/ OR exp Equatorial Guinea/ OR exp Eritrea/ OR exp Ethiopia/ OR exp Gabon/ OR exp Gambia/ OR exp Ghana/ OR exp Guinea-Bissau/ OR exp Guinea/ OR exp Kenya/ OR exp Lesotho/ OR exp Liberia/ OR exp Libya/ OR exp Malawi/ OR exp Mali/ OR exp Mauritania/ OR exp Morocco/ OR exp Mozambique/ OR exp Namibia/ OR exp Niger"/ OR exp Nigeria/ OR exp Rwanda/ OR exp Senegal/ OR exp Sierra Leone/ OR exp Somalia/ OR exp South Africa/ OR exp South Sudan/ OR exp Sudan/ OR exp Swaziland/ OR exp Tanzania/ OR exp Togo/ OR exp Tunisia/ OR exp Uganda/ OR exp Zambia/ OR exp Zimbabwe/
#4	#2 OR #3
#5	(randomized controlled trial[pt] OR controlled clinical trial[pt] OR randomized[tiab] OR placebo[tiab] OR clinical trials as topic[mesh:noexp] OR randomly[tiab] OR trial[ti] NOT (animals[mh] NOT humans [mh]))
#6	#1 AND #4 AND #5

Embase – search string

1. (((singl* or doubl* or treb* or tripl*) adj (blind*3 or mask$3)) or (allocated adj2 random)).tw. or (clin* adj25 trial*).ti,ab. or (clinic: adj trial$1).tw. or (double-blind* or random*).af. or exp "clinical trial (topic)"/ or exp double blind procedure/ or exp single blind procedure/ or exp triple blind procedure/ or placebo*.tw. or exp placebo/ or exp randomization/ or Random.af. or Random*.tw. or exp "randomized controlled trial (topic)"/ or randomized.ab. or randomly allocated.tw. or randomly.ab. or trial.ab. or trial.ti. or exp "controlled clinical trial (topic)"/ or randomized controlled trial/ or "randomized controlled trial (topic)"/ or exp controlled clinical trial/
2. Africa* or North Africa* or northern Africa* or Africa south of the sahara or central Africa* or south Africa* or southern Africa* or north Africa* or northern Africa* or east Africa* or eastern Africa* or west Africa* or western Africa* or Algeria* or Angola* or Benin* or Botswana or Botswanese or Burkina Faso or Burundi* or Cameroon* or Cape Verde or Cape Verdean or Central African republic or Chad* or Congo* or democratic republic congo or Cote d'ivoire or Ivorian or Comoros or Comoran or Djibouti* or Egypt* or Equatorial Guinea* or Eritrea* or Ethiopia* or Gabon* or Gambia* or Ghana* or Guinea-Bissau or Bisseau-Guinean or Guinea* or Kenya* or Lesotho or Basotho or Mosotho or Liberia* or Libya* or libyan arab jamahiriya or Madagascar or Madagascan or Malawi* or Mali* or Mayotte or Mauritania* or Morocco or Moroccan or Mozambique or Mozambicans or Namibia* or Niger* or Nigeria* or Rwanda* or Senegal* or Sengalese or Sierra leone* or Somalia* or South Sudan* or Sudan* or Swaziland or Swazis or Tanzania* or Togo* or Tunisia* or Uganda* or Zambia* or Zimbabwe*
3. "africa south of the sahara"/ or angola/ or benin/ or botswana/ or burkina faso/ or burundi/ or cameroon/ or cape verde/ or central africa/ or central african republic/ or chad/ or comoros/ or congo/ or cote d'ivoire/ or democratic republic congo/ or djibouti/ or equatorial guinea/ or eritrea/ or ethiopia/ or gabon/ or gambia/ or ghana/ or guinea/ or guinea-bissau/ or kenya/ or lesotho/ or liberia/ or madagascar/ or malawi/ or mali/ or mayotte/ or mozambique/ or namibia/ or niger/ or nigeria/ or rwanda/ or senegal/ or sierra leone/ or somalia/ or south africa/ or south sudan/ or sudan/ or swaziland/ or tanzania/ or togo/ or uganda/ or zambia/ or zimbabwe/ or north africa/ or north african/ or algeria/ or egypt/ or libyan arab jamahiriya/ or mauritania/ or morocco/ or tunisia/ or western sahara/ or Central Africa/ or central african/ or north african/ or South Africa/ or southern african/ or african/ or west african/ or exp Africa/
4. 2. Or 3
5. cell phone based or cell phone communication or cell phone intervention* or cell phone reminder* or cell phone service* or cell phone text messag* or health app* or health communication or ipad* or mobile application* or mhealth or mobile health or mobile health application* or mobile health care or mobile healthcare or mobile health intervention* or mobile phone messag* or mobile phone reminder* or mobile phone short message or short message service* or short messaging service* or smartphone or smart-phone or sms or sms intervention* or sms messag* or sms reminder* or sms-reminders or text messag* or text message based or text message intervention* or text message reminder* or texting or two way sms or two-way sms or two way text messag* or two-way text messag* or interactive sms or interactive text messag*
6. telemedicine/ or mobile application/ or mobile phone/ or text messaging/ or reminder system/
7. 5 or 6
8. 1 and 4 and 7

Cochrane Central Register of systematic trials – search string

#1	MeSH descriptor: [Africa] explode all trees
#2	MeSH descriptor: [Africa, Northern] explode all trees
#3	MeSH descriptor: [Africa, Central] explode all trees
#4	MeSH descriptor: [Africa, Eastern] explode all trees
#5	MeSH descriptor: [Africa, Western] explode all trees
#6	MeSH descriptor: [Africa, Southern] explode all trees

#7	MeSH descriptor: [Africa South of the Sahara] explode all trees
#8	#1 or #2 or #3 or #4 or #5 or #6 or #7
#9	(("Africa South of the Sahara" or "Africa, Eastern") or "Africa, Northern") or "Africa, Southern") or "Africa, Western") or "Africa") or "African") or "Algeria") or "Angola") or "Benin") or "Botswana") or "Burkina Faso") or "Burundi") or "Cameroon") or "Cape Verde") or "Central African Republic") or "Chad") or "Congo") or "Cote d'Ivoire") or "Democratic Republic of the Congo") or "Djibouti") or "Egypt") or "Equatorial Guinea") or "Eritrea") or "Ethiopia") or "Gabon") or "Gambia") or "Ghana") or "Guinea-Bissau") or "Guinea") or "Kenya") or "Lesotho") or "Liberia") or "Libya") or "Malawi") or "Mali") or "Mauritania") or "Morocco") or "Mozambique") or "Namibia") or "Niger") or "Nigeria") or "Rwanda") or "Senegal") or "Sierra Leone") or "Somalia") or "South Africa") or "South Sudan") or "Sudan") or "Swaziland") or "Tanzania") or "Togo") or "Tunisia") or "Uganda") or "Zambia") or "Zimbabwe"
#10	#8 or #9
#12	MeSH descriptor: [Telemedicine] 2 tree(s) exploded
#13	MeSH descriptor: [Mobile Applications] explode all trees
#14	MeSH descriptor: [Cell Phone] explode all trees
#15	MeSH descriptor: [Smartphone] explode all trees
#16	MeSH descriptor: [Text Messaging] explode all trees
#17	MeSH descriptor: [Reminder Systems] explode all trees
#18	MeSH descriptor: [Health Communication] explode all trees
#19	#12 or #13 or #14 or #15 or #16 or #17 or #18
#20	cell phone based
#21	((("cell phone based" or "cell phone communication") or "cell phone intervention") or "cell phone interventions") or "cell phone reminders") or "cell phone service") or "cell phone text message") or "cell phone text messages") or "cell phone text messaging") or "health app") or "health apps") or "health application") or "health applications") or "health communication") or ipad) or pads) or "mobile application") or "mobile applications") or mHealth) or "mobile health") or "mobile health application") or "mobile health applications") or "mobile health care") or "mobile healthcare") or "mobile health intervention") or "mobile health interventions") or "mobile phone application") or "mobile phone applications") or "mobile phone call") or "mobile phone calls") or "mobile phone communication") or "mobile phone communications") or "mobile phone intervention") or "mobile phone interventions") or "mobile phone message") or "mobile phone messages") or "mobile phone messaging") or "mobile phone reminder") or "mobile phone reminders") or "mobile phone short message") or "short message service") or "short message services") or "short messaging service") or "short messaging services") or smartphone) or smart-phone) or sms) or "sms intervention") or "sms interventions") or "sms message") or "sms messages") or "sms messaging") or "sms reminder") or "sms-reminder") or "sms reminders") or "sms-reminders") or "text message") or "text messages") or "text messaging") or "text message based") or "text message intervention") or "text message interventions") or "text message reminder") or "text message reminders") or "text message-reminder") or "text message-reminders") or texting
#22	#20 or #21
#23	#22 and #10

The Global Health Library – search string

S20	S6 AND S15 AND S19
S19	S16 OR S17 OR S18
S18	(("cell phone based") OR "cell phone communication") OR "cell phone intervention") OR "cell phone interventions") OR "cell phone reminders") OR "cell phone service") OR "cell phone text message") OR "cell phone text messages") OR "cell phone text messaging") OR "health app") OR "health apps") OR "health application") OR "health applications") OR "health communication") OR ipad) OR pads) OR "mobile application") OR "mobile applications") OR mHealth) OR "mobile health") OR "mobile health application") OR "mobile health applications") OR "mobile health care") OR "mobile healthcare") OR "mobile health intervention") OR "mobile health interventions") OR "mobile phone application") OR "mobile phone applications") OR "mobile phone call") OR "mobile phone calls") OR "mobile phone communication") OR "mobile phone communications") OR "mobile phone intervention") OR "mobile phone interventions") OR "mobile phone message") OR "mobile phone messages") OR "mobile phone messaging") OR "mobile phone reminder") OR "mobile phone reminders") OR "mobile phone short message") OR "short message service") OR "short message services") OR "short messaging service") OR "short messaging services") OR smartphone) OR smart-phone) OR sms) OR "sms intervention") OR "sms interventions") OR "sms message") OR "sms messages") OR "sms messaging") OR "sms reminder") OR "sms-reminder") OR "sms reminders") OR "sms-reminders") OR "text message") OR "text messages") OR "text messaging") OR "text message based") OR "text message intervention") OR "text message interventions") OR "text message reminder") OR "text message reminders") OR "text message-reminder") OR "text message-reminders") OR «texting”) OR “two way SMS”) OR “two way SMS”) OR “two-way SMS”) OR “two-way text message”) OR “interactive SMS”) OR “interactive text message
S17	DE "telecommunications"
S16	DE "telephones"
S15	S13 OR S14
S14	((("Africa South of the Sahara") OR "Africa, Eastern") OR "Africa, Northern") OR "Africa, Southern") OR "Africa, Western") OR "Africa") OR “African”) OR "Algeria") OR "Angola") OR "Benin") OR "Botswana") OR "Burkina Faso") OR "Burundi") OR "Cameroon") OR "Cape Verde") OR "Central African Republic") OR "Chad") OR "Congo") OR "Cote d'Ivoire") OR "Democratic Republic of the Congo") OR "Djibouti") OR "Egypt") OR "Equatorial Guinea") OR "Eritrea") OR "Ethiopia") OR "Gabon") OR "Gambia") OR "Ghana") OR "Guinea-Bissau") OR "Guinea") OR "Kenya") OR "Lesotho") OR "Liberia") OR "Libya") OR "Malawi") OR "Mali") OR "Mauritania") OR "Morocco") OR "Mozambique") OR "Namibia") OR "Niger") OR "Nigeria") OR "Rwanda") OR "Senegal") OR "Sierra Leone") OR "Somalia") OR "South Africa") OR "South Sudan") OR "Sudan") OR "Swaziland") OR "Tanzania") OR "Togo") OR "Tunisia") OR "Uganda") OR "Zambia") OR "Zimbabwe"
S13	S7 OR S8 OR S9 OR S10 OR S11 OR S12
S12	DE "Africa"
S11	DE "Egypt" OR DE "Libya" OR DE "Maghreb"
S10	DE "Southern Africa" OR DE "Botswana" OR DE "Comoros" OR DE "Lesotho" OR DE "Mozambique" OR DE "Namibia" OR DE "Saint Helena" OR DE "South Africa" OR DE "Swaziland" OR DE "Angola" OR DE "Zambia" OR DE "Zimbabwe"
S9	DE "West Africa" OR DE "Benin" OR DE "Burkina Faso" OR DE "Cape Verde" OR DE "Cote d'Ivoire" OR DE "Gambia" OR DE "Ghana" OR DE "Guinea" OR DE "Guinea-Bissau" OR DE "Liberia" OR DE "Mali" OR DE "Mauritania" OR DE "Niger" OR DE "Nigeria" OR DE "Senegal" OR DE "Sierra Leone" OR DE "Togo" OR DE "Western Sahara" OR DE "Sahel"
S8	DE "East Africa" OR DE "Djibouti" OR DE "Eritrea" OR DE "Ethiopia" OR DE "Kenya" OR DE "Madagascar" OR DE "Malawi" OR DE "Rwanda" OR DE "Seychelles" OR DE "Somalia" OR DE "Sudan" OR DE "Tanzania" OR DE "Uganda"

S7	DE "Central Africa" OR DE "Africa South of Sahara" OR DE "Burundi" OR DE "Cameroon" OR DE "Central African Republic" OR DE "Chad" OR DE "Congo" OR DE "Congo Democratic Republic" OR DE "Equatorial Guinea" OR DE "Gabon" OR DE "Sao Tome and Principe"
S6	S1 OR S2 OR S3 OR S4 OR S5
S5	AB "random allocation" OR AB "random allocations" OR AB "random allocated" OR AB "randomly allocated" OR AB "clinical trials" OR AB placebo OR AB placebos OR AB "drug therapy" OR "review of reported cases" OR "multicase review" OR "practice guideline"
S4	"randomized controlled trial" OR "Clinical Trial" OR "Controlled Clinical Trial" OR AB "single blind" OR AB "single blinded" OR AB "single masked" OR AB "double blind" OR AB "double blinded" OR AB "double masked" OR AB "triple blind" OR "triple blinded" OR "triple masked"
S3	DE "reviews"
S2	DE "placebos"
S1	DE "randomized controlled trials" OR DE "clinical trials"
