# Supplementary material for: Two-way text message interventions and healthcare outcomes in Africa: Systematic review of randomized trials with meta-analyses on appointment attendance and medicine adherence
Source: PLoS One. 2022 Apr 14;17(4):e0266717. doi: 10.1371/journal.pone.0266717 (PMC9009629; doi:10.1371/journal.pone.0266717)
Supplement: S3 File — (PDF) [file pone.0266717.s003.pdf]

S3 File: Dataset used for meta-analysis

Name	Data Type	Statistical Method	Effect Measure	Analysis Model	Events	Non-event	Total 1	Events 2	Non-events 2	Total 2	Effect Estimate	SE	CI Start	CI End	Weight	Q	P(Q)	I-Sqr(Q)	Tau-Sqr	Z	P(Z)	Qint	P(Qint)	I-Sqr(Qint)	df
**Inverse variance**																									
**AA_primary analysis (AA=appointment attendance)**	**INV**	**IV**	**Risk Ratio**	**Random**			**2082**			**2292**	**103.335.299**		**0.95137946**	**11.223.896**	**100**	**855.247.198**	**0.07331272**	**5.322.989.647**	**0.00378164**	**0.77801915**	**0.43655773**	**0**	**1**	**0**	**4**
Bobrow 2016*					378	80	458	383	74	457	0.98481645	0.0297	0.92912599	104.384.492	3.813.009.758										
Leiby 2016					67	466	533	57	493	550	121.288.279	0.1696	0.8698714	16.911.519	546.394.681										
Odeny 2014					38	156	194	22	165	187	166.495.817	0.2475	102.501.671	270.442.978	273.422.959										
Odeny 2019					213	335	548	259	488	747	111.996.787	0.0734	0.96990092	129.325.378	1.939.411.322										
Van der Kop 2018					277	72	349	285	66	351	0.97745796	0.0375	0.90819274	105.200.582	3.427.761.281										
**AA_sensitivity (+Lund)**	**INV**	**IV**	**Risk Ratio**	**Random**			**3393**			**3531**	**114.632.226**		**0.99099948**	**132.598.932**	**100**	**4.450.882.966**	**0.00000002**	**8.876.627.393**	**0.02406663**	**183.825.691**	**0.06602456**	**0**	**1**	**0**	**5**
Bobrow 2016					378	80	458	383	74	457	0.98481645	0.0297	0.92912599	104.384.492	2.211.966.872										
Leiby 2016					67	466	533	57	493	550	121.288.279	0.1696	0.8698714	16.911.519	1.044.575.368										
Lund 2014					574	737	1311	385	854	1239	140.902.785	0.0526	127.100.242	156.204.226	2.056.607.164										
Odeny 2014					38	156	194	22	165	187	166.495.817	0.2475	102.501.671	270.442.978	646.787.143										
Odeny 2019					213	335	548	259	488	747	111.996.787	0.0734	0.96990092	129.325.378	1.873.612.619										
Van der Kop 2018					277	72	349	285	66	351	0.97745796	0.0375	0.90819274	105.200.582	2.166.450.834										
**AA_subgroup (intervention type)**	**INV**	**IV**	**Risk Ratio**	**Random**			**2082**			**2292**	**103.335.299**		**0.95137946**	**11.223.896**	**100**	**855.247.198**	**0.07331272**	**5.322.989.647**	**0.00378164**	**0.77801915**	**0.43655773**	**187.029.564**	**0.17144142**	**4.653.251.719**	**4**
**No HCP contact**	**INV**	**IV**	**Risk Ratio**	**Random**			**1340**			**1358**	**0.98581685**		**0.94224182**	**103.140.706**	**7.787.165.719**	**154.649.832**	**0.46151124**	**0**	**0**	**0.61929504**	**0.53572201**				**2**
Bobrow 2016					378	80	458	383	74	457	0.98481645	0.0297	0.92912599	104.384.492	3.813.009.758										
Leiby 2016					67	466	533	57	493	550	121.288.279	0.1696	0.8698714	16.911.519	546.394.681										
Van der Kop 2018					277	72	349	285	66	351	0.97745796	0.0375	0.90819274	105.200.582	3.427.761.281										
**HCP contact**	**INV**	**IV**	**Risk Ratio**	**Random**			**742**			**934**	**127.264.534**		**0.88505822**	**182.996.568**	**2.212.834.281**	**235.899.253**	**0.12456277**	**576.090.222**	**0.04528422**	**130.105.555**	**0.19323944**				**1**
Odeny 2014					38	156	194	22	165	187	166.495.817	0.2475	102.501.671	270.442.978	273.422.959										
Odeny 2019					213	335	548	259	488	747	111.996.787	0.0734	0.96990092	129.325.378	1.939.411.322										
**AA_sensitivity (-cluster)**	**INV**	**IV**	**Risk Ratio**	**Random**			**1534**			**1545**	**101.007.773**		**0.92647165**	**110.122.854**	**100**	**599.175.409**	**0.11201215**	**4.993.118.954**	**0.00314766**	**0.2274691**	**0.82005899**	**0**	**1**	**0**	**3**
Bobrow 2016					378	80	458	383	74	457	0.98481645	0.0297	0.92912599	104.384.492	4.822.190.073										
Leiby 2016					67	466	533	57	493	550	121.288.279	0.1696	0.8698714	16.911.519	608.934.911										
Odeny 2014					38	156	194	22	165	187	166.495.817	0.2475	102.501.671	270.442.978	301.724.248										
Van der Kop 2018					277	72	349	285	66	351	0.97745796	0.0375	0.90819274	105.200.582	4.267.150.768										
**AA_sensitivty (fixed effect)**	**INV**	**IV**	**Risk Ratio**	**Fixed**			**2130**			**2292**	**10.010.906**		**0.9589312**	**104.510.354**	**100**	**85.817.576**	**0.07244755**	**5.338.950.148**	**0**	**0.04965304**	**0.96039888**	**0**	**1**	**0**	**4**
Van der Kop 2018					277	72	349	285	66	351	0.97745796	0.0375	0.90819274	105.200.582	3.426.907.382										
Bobrow 2016					378	80	458	383	74	457	0.98481645	0.0297	0.92912599	104.384.492	5.463.261.692										
Odeny 2019					213	335	548	259	488	747	112.299.587	0.0747	0.9700484	130.005.856	863.622.004										
Leiby 2016					67	466	533	57	493	550	121.288.279	0.1696	0.8698714	16.911.519	167.537.954										
Odeny 2014					38	156	194	22	165	187	166.495.817	0.2475	102.501.671	270.442.978	0.78670968										
**AA_subgroup (Rob)**	**INV**	**IV**	**Risk Ratio**	**Random**			**2082**			**2292**	**103.335.299**		**0.95137946**	**11.223.896**	**100**	**855.247.198**	**0.07331272**	**5.322.989.647**	**0.00378164**	**0.77801915**	**0.43655773**	**204.086.835**	**0.15312264**	**5.100.124.905**	**4**
**Low risk of bias**	**INV**	**IV**	**Risk Ratio**	**Random**			**349**			**351**	**0.97745796**		**0.90819274**	**105.200.582**	**3.427.761.281**	**0**	**0**	**100**	**0**	**0.608**	**0.54318748**				**0**
Van der Kop 2018					277	72	349	285	66	351	0.97745796	0.0375	0.90819274	105.200.582	3.427.761.281										
**High risk of bias**	**INV**	**IV**	**Risk Ratio**	**Random**			**1733**			**1941**	**110.362.132**		**0.95041192**	**128.152.856**	**6.572.238.719**	**793.017.897**	**0.04747705**	**6.216.983.233**	**0.01213422**	**129.299.237**	**0.1960137**				**3**
Bobrow 2016					378	80	458	383	74	457	0.98481645	0.0297	0.92912599	104.384.492	3.813.009.758										
Leiby 2016					67	466	533	57	493	550	121.288.279	0.1696	0.8698714	16.911.519	546.394.681										
Odeny 2014					38	156	194	22	165	187	166.495.817	0.2475	102.501.671	270.442.978	273.422.959										
Odeny 2019					213	335	548	259	488	747	111.996.787	0.0734	0.96990092	129.325.378	1.939.411.322										
**AA_subgroup (clininical area)**	**INV**	**IV**	**Risk Ratio**	**Random**			**2082**			**2292**	**103.335.299**		**0.95137946**	**11.223.896**	**100**	**855.247.198**	**0.07331272**	**5.322.989.647**	**0.00378164**	**0.77801915**	**0.43655773**	**186.525.591**	**0.17201961**	**4.638.805.345**	**4**
**Hypertension**	**INV**	**IV**	**Risk Ratio**	**Random**			**458**			**457**	**0.98481645**		**0.92912599**	**104.384.492**	**3.813.009.758**	**0**	**1**	**0**	**0**	**0.51515152**	**0.60644715**				**0**
Bobrow 2016					378	80	458	383	74	457	0.98481645	0.0297	0.92912599	104.384.492	3.813.009.758										
**HIV**	**INV**	**IV**	**Risk Ratio**	**Random**			**1624**			**1835**	**110.464.916**		**0.94682932**	**128.877.479**	**6.186.990.242**	**787.443.753**	**0.04867934**	**6.190.204.076**	**0.01295058**	**126.534.464**	**0.20574784**				**3**
Leiby 2016					67	466	533	57	493	550	121.288.279	0.1696	0.8698714	16.911.519	546.394.681										
Odeny 2014					38	156	194	22	165	187	166.495.817	0.2475	102.501.671	270.442.978	273.422.959										
Odeny 2019					213	335	548	259	488	747	111.996.787	0.0734	0.96990092	129.325.378	1.939.411.322										
Van der Kop 2018					277	72	349	285	66	351	0.97745796	0.0375	0.90819274	105.200.582	3.427.761.281										
**AA-sens-1way2way**	**INV**	**IV**	**Risk Ratio**	**Random**			**458**			**457**	**0.9952115**		**0.93801352**	**105.589.728**	**100**	**0**	**1**	**0**	**0**	**0.1589404**	**0.87371583**	**0**	**1**	**0**	**0**
Bobrow 2016					378	80	458	383	74	457	0.9952115	0.0302	0.93801352	105.589.728	100										
**MA_primary analysis (MA=Medicine adherence)**	**INV**	**IV**	**Risk Ratio**	**Random**			**1405**			**1378**	**113.879.514**		**106.879.897**	**121.337.539**	**100**	**543.576.454**	**0.36504104**	**801.661.916**	**0.00050986**	**401.571.881**	**0.00005926**	**0**	**1**	**0**	**5**
Bobrow 2016					225	233	458	190	267	457	11.816.361	0.073	102.410.865	136.339.426	1.794.058.574										
Kassaye 2016					174	106	280	162	108	270	103.572.328	0.0682	0.90613267	118.384.728	2.029.655.633										
Lester 2010					165	108	273	132	133	265	121.324.671	0.0785	104.023.102	141.503.911	1.570.006.782										
Mbuagbaw 2012					72	29	101	69	30	99	102.285.731	0.0915	0.85492913	122.377.055	1.179.366.149										
Modrek 2014					170	43	213	135	71	206	121.786.582	0.0612	108.020.432	137.307.094	2.461.695.666										
Sumari-De Boer 2021					60	20	80	54	27	81	112.501.909	0.1017	0.92170659	137.317.879	965.217.196										
**MA_sensitivity (-cluster)**	**INV**	**IV**	**Risk Ratio**	**Random**			**1125**			**1108**	**116.804.747**		**109.136.237**	**125.012.087**	**100**	**296.533.289**	**0.56364358**	**0**	**0**	**448.332.792**	**0.00000735**	**0**	**1**	**0**	**4**
Bobrow 2016					225	233	458	190	267	457	11.816.361	0.073	102.410.865	136.339.426	2.252.597.752										
Lester 2010					165	108	273	132	133	265	121.324.671	0.0785	104.023.102	141.503.911	1.948.004.937										
Mbuagbaw 2012					72	29	101	69	30	99	102.285.731	0.0915	0.85492913	122.377.055	1.433.795.386										
Modrek 2014					170	43	213	135	71	206	121.786.582	0.0612	108.020.432	137.307.094	3.204.988.845										
Sumari-De Boer 2021					60	20	80	54	27	81	112.501.909	0.1017	0.92170659	137.317.879	116.061.308										
**MA_sensitiviy (fixed effect)**	**INV**	**IV**	**Risk Ratio**	**Fixed**			**1405**			**1378**	**113.959.026**		**107.264.397**	**121.071.482**	**100**	**543.576.454**	**0.36504104**	**801.661.916**	**0**	**423.020.985**	**0.00002335**	**0**	**1**	**0**	**5**
Bobrow 2016					225	233	458	190	267	457	11.816.361	0.073	102.410.865	136.339.426	1.790.499.081										
Kassaye 2016					174	106	280	162	108	270	103.572.328	0.0682	0.90613267	118.384.728	2.051.403.411										
Lester 2010					165	108	273	132	133	265	121.324.671	0.0785	104.023.102	141.503.911	1.548.390.539										
Mbuagbaw 2012					72	29	101	69	30	99	102.285.731	0.0915	0.85492913	122.377.055	1.139.666.111										

Modrek 2014					170	43	213	135	71	206	121.786.582	0.0612	108.020.432	137.307.094	254.751.634										
Sumari-De Boer 2021					60	20	80	54	27	81	112.501.909	0.1017	0.92170659	137.317.879	922.524.517										
**MA_subgroup (Rob)**	**INV**	**IV**	**Risk Ratio**	**Random**			**1405**			**1378**	**113.879.514**		**106.879.897**	**121.337.539**	**100**	**543.576.454**	**0.36504104**	**801.661.916**	**0.00050986**	**401.571.881**	**0.00005926**	**157.437.502**	**0.20957284**	**3.648.273.193**	**5**
**Low risk of bias**	**INV**	**IV**	**Risk Ratio**	**Random**			**101**			**99**	**102.285.731**		**0.85492913**	**122.377.055**	**1.179.366.149**	**0**	**1**	**0**	**0**	**0.24699454**	**0.80491245**				**0**
Mbuagbaw 2012					72	29	101	69	30	99	102.285.731	0.0915	0.85492913	122.377.055	1.179.366.149										
**High risk of bias**	**INV**	**IV**	**Risk Ratio**	**Random**			**1304**			**1279**	**115.554.165**		**108.355.915**	**123.230.605**	**8.820.633.851**	**386.138.952**	**0.42508977**	**0**	**0**	**440.545.617**	**0.00001056**				**4**
Bobrow 2016					225	233	458	190	267	457	11.816.361	0.073	102.410.865	136.339.426	1.794.058.574										
Kassaye 2016					174	106	280	162	108	270	103.572.328	0.0682	0.90613267	118.384.728	2.029.655.633										
Lester 2010					165	108	273	132	133	265	121.324.671	0.0785	104.023.102	141.503.911	1.570.006.782										
Modrek 2014					170	43	213	135	71	206	121.786.582	0.0612	108.020.432	137.307.094	2.461.695.666										
Sumari-De Boer 2021					60	20	80	54	27	81	112.501.909	0.1017	0.92170659	137.317.879	965.217.196										
**MA_subgroup (clinical area)**	**INV**	**IV**	**Risk Ratio**	**Random**			**1405**			**1378**	**113.879.514**		**106.879.897**	**121.337.539**	**100**	**543.576.454**	**0.36504104**	**801.661.916**	**0.00050986**	**401.571.881**	**0.00005926**	**243.906.221**	**0.2953687**	**1.800.127.162**	**5**
**Hypertension**	**INV**	**IV**	**Risk Ratio**	**Random**			**458**			**457**	**11.816.361**		**102.410.865**	**136.339.426**	**1.794.058.574**	**0**	**1**	**0**	**0**	**228.630.137**	**0.02223663**				**0**
Bobrow 2016					225	233	458	190	267	457	11.816.361	0.073	102.410.865	136.339.426	1.794.058.574										
**HIV**	**INV**	**IV**	**Risk Ratio**	**Random**			**734**			**715**	**109.343.234**		**100.890.225**	**118.504.471**	**574.424.576**	**299.670.233**	**0.39213409**	**0**	**0**	**21.758.619**	**0.02956559**				**3**
Kassaye 2016					174	106	280	162	108	270	103.572.328	0.0682	0.90613267	118.384.728	2.029.655.633										
Lester 2010					165	108	273	132	133	265	121.324.671	0.0785	104.023.102	141.503.911	1.570.006.782										
Mbuagbaw 2012					72	29	101	69	30	99	102.285.731	0.0915	0.85492913	122.377.055	1.179.366.149										
Sumari-De Boer 2021					60	20	80	54	27	81	112.501.909	0.1017	0.92170659	137.317.879	965.217.196										
**Malaria**	**INV**	**IV**	**Risk Ratio**	**Random**			**213**			**206**	**121.786.582**		**108.020.432**	**137.307.094**	**2.461.695.666**	**0**	**1**	**0**	**0**	**322.058.824**	**0.00127928**				**0**
Modrek 2014					170	43	213	135	71	206	121.786.582	0.0612	108.020.432	137.307.094	2.461.695.666										
**MA_subgroup (intervention type)**	**INV**	**IV**	**Risk Ratio**	**Random**			**1405**			**1378**	**113.879.514**		**106.879.897**	**121.337.539**	**100**	**543.576.454**	**0.36504104**	**801.661.916**	**0.00050986**	**401.571.881**	**0.00005926**	**491.501.235**	**0.02662441**	**7.965.417.116**	**5**
**No HCP contact**	**INV**	**IV**	**Risk Ratio**	**Random**			**1024**			**1009**	**119.428.802**		**110.980.043**	**128.520.754**	**6.790.978.217**	**0.50875464**	**0.91696395**	**0**	**0**	**474.297.388**	**0.00000211**				**3**
Bobrow 2016					225	233	458	190	267	457	11.816.361	0.073	102.410.865	136.339.426	1.794.058.574										
Lester 2010					165	108	273	132	133	265	121.324.671	0.0785	104.023.102	141.503.911	1.570.006.782										
Modrek 2014					170	43	213	135	71	206	121.786.582	0.0612	108.020.432	137.307.094	2.461.695.666										
Sumari-De Boer 2021					60	20	80	54	27	81	112.501.909	0.1017	0.92170659	137.317.879	965.217.196										
**HCP contact**	**INV**	**IV**	**Risk Ratio**	**Random**			**381**			**369**	**103.110.982**		**0.92631736**	**114.775.726**	**3.209.021.783**	**0.01199755**	**0.91277948**	**0**	**0**	**0.56025575**	**0.57530501**				**1**
Kassaye 2016					174	106	280	162	108	270	103.572.328	0.0682	0.90613267	118.384.728	2.029.655.633										
Mbuagbaw 2012					72	29	101	69	30	99	102.285.731	0.0915	0.85492913	122.377.055	1.179.366.149										
**MA_2-way vs. 1-way**	**INV**	**IV**	**Risk Ratio**	**Random**			**562**			**562**	**0.89228341**		**0.79593039**	**100.030.065**	**100**	**0.29514855**	**0.58694003**	**0**	**0**	**195.480.817**	**0.05060572**	**0**	**1**	**0**	**1**
Abiodun 2021					47	57	104	57	48	105	0.83251836	0.1403	0.63236852	109.601.727	1.726.905.926										
Bobrow 2016					225	233	458	190	267	457	0.90528995	0.0641	0.79840947	102.647.817	8.273.094.074										
*data provided by primary author via email - not stated in article																									
