# Supplementary material for: Two-way text message interventions and healthcare outcomes in Africa: Systematic review of randomized trials with meta-analyses on appointment attendance and medicine adherence
Source: PLoS One. 2022 Apr 14;17(4):e0266717. doi: 10.1371/journal.pone.0266717 (PMC9009629; doi:10.1371/journal.pone.0266717)
Supplement: S4 File — (PDF) [file pone.0266717.s004.pdf]

S4 File: Subgroup and sensitivity analyses

Study or Subgroup	log[Risk Ratio]	SE	2-way text message Total	1-way text message Total	Weight	Risk Ratio IV, Random, 95% CI
Bobrow 2016	−0.0048	0.0302	458	457	100.0%	1.00 [0.94, 1.06]
**Total (95% CI)**			**458**	**457**	**100.0%**	**1.00 [0.94, 1.06]**

Heterogeneity: Not applicable
Test for overall effect: Z = 0.16 (P = 0.87)

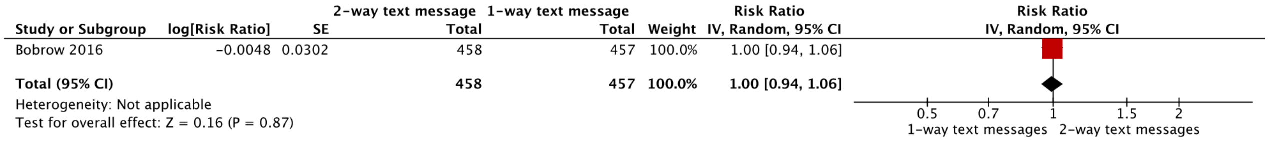

Figure A: Two-way text messages versus one-way text messages on appointment attendance

Study or Subgroup	log[Risk Ratio]	SE	2-way text message Total	1-way text message Total	Weight	Risk Ratio IV, Random, 95% CI
Abiodun 2021	−0.1833	0.1403	104	105	17.3%	0.83 [0.63, 1.10]
Bobrow 2016	−0.0995	0.0641	458	457	82.7%	0.91 [0.80, 1.03]
**Total (95% CI)**			**562**	**562**	**100.0%**	**0.89 [0.80, 1.00]**

Heterogeneity: Tau^2 = 0.00; Chi^2 = 0.30, df = 1 (P = 0.59); I^2 = 0%
Test for overall effect: Z = 1.95 (P = 0.05)

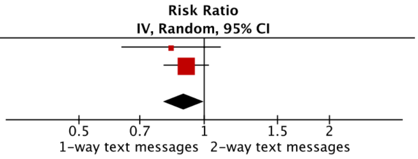

Figure B: Two-way text messages versus one-way text messages on medicine adherence

Study or Subgroup	log[Risk Ratio]	SE	2-way text messages Total	Standard care Total	Weight	Risk Ratio IV, Random, 95% CI
**5.6.2 Low risk of bias**						
Van der Kop 2018	−0.0228	0.0375	349	351	34.3%	0.98 [0.91, 1.05]
**Subtotal (95% CI)**			**349**	**351**	**34.3%**	**0.98 [0.91, 1.05]**
Heterogeneity: Not applicable						
Test for overall effect: Z = 0.61 (P = 0.54)						
**5.6.3 High risk of bias**						
Bobrow 2016	−0.0153	0.0297	458	457	38.1%	0.98 [0.93, 1.04]
Leiby 2016	0.193	0.1696	533	550	5.5%	1.21 [0.87, 1.69]
Odeny 2014	0.5098	0.2475	194	187	2.7%	1.66 [1.03, 2.70]
Odeny 2019	0.1133	0.0734	548	747	19.4%	1.12 [0.97, 1.29]
**Subtotal (95% CI)**			**1733**	**1941**	**65.7%**	**1.10 [0.95, 1.28]**
Heterogeneity: Tau^2 = 0.01; Chi^2 = 7.93, df = 3 (P = 0.05); I^2 = 62%						
Test for overall effect: Z = 1.29 (P = 0.20)						
**Total (95% CI)**			**2082**	**2292**	**100.0%**	**1.03 [0.95, 1.12]**

Heterogeneity: Tau^2 = 0.00; Chi^2 = 8.55, df = 4 (P = 0.07); I^2 = 53%
Test for overall effect: Z = 0.78 (P = 0.44)
Test for subgroup differences: Chi^2 = 2.04, df = 1 (P = 0.15), I^2 = 51.0%

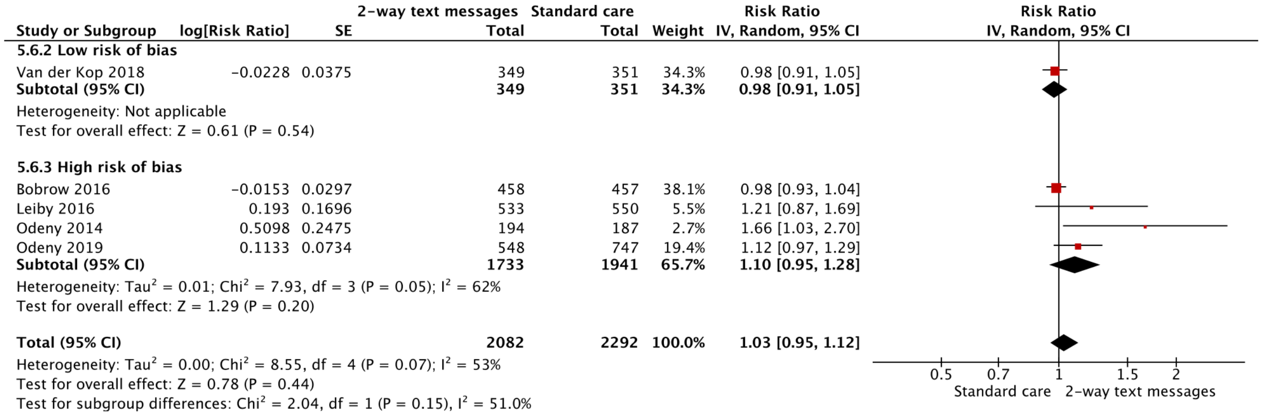

Figure C: Subgroup analysis: Low risk of bias versus high risk of bias on appointment attendance

Study or Subgroup	log[Risk Ratio]	SE	2-way text messages Total	Standard care Total	Weight	Risk Ratio IV, Random, 95% CI
**5.14.1 Low risk of bias**						
Mbuagbaw 2012	0.0226	0.0915	101	99	11.8%	1.02 [0.85, 1.22]
**Subtotal (95% CI)**			**101**	**99**	**11.8%**	**1.02 [0.85, 1.22]**
Heterogeneity: Not applicable						
Test for overall effect: Z = 0.25 (P = 0.80)						
**5.14.2 High risk of bias**						
Bobrow 2016	0.1669	0.073	458	457	17.9%	1.18 [1.02, 1.36]
Kassaye 2016	0.0351	0.0682	280	270	20.3%	1.04 [0.91, 1.18]
Lester 2010	0.1933	0.0785	273	265	15.7%	1.21 [1.04, 1.42]
Modrek 2014	0.1971	0.0612	213	206	24.6%	1.22 [1.08, 1.37]
Sumari–De Boer 2021	0.1178	0.1017	80	81	9.7%	1.13 [0.92, 1.37]
**Subtotal (95% CI)**			**1304**	**1279**	**88.2%**	**1.16 [1.08, 1.23]**
Heterogeneity: Tau^2 = 0.00; Chi^2 = 3.86, df = 4 (P = 0.43); I^2 = 0%						
Test for overall effect: Z = 4.41 (P < 0.0001)						
**Total (95% CI)**			**1405**	**1378**	**100.0%**	**1.14 [1.07, 1.21]**

Heterogeneity: Tau^2 = 0.00; Chi^2 = 5.44, df = 5 (P = 0.37); I^2 = 8%
Test for overall effect: Z = 4.02 (P < 0.0001)
Test for subgroup differences: Chi^2 = 1.57, df = 1 (P = 0.21), I^2 = 36.5%

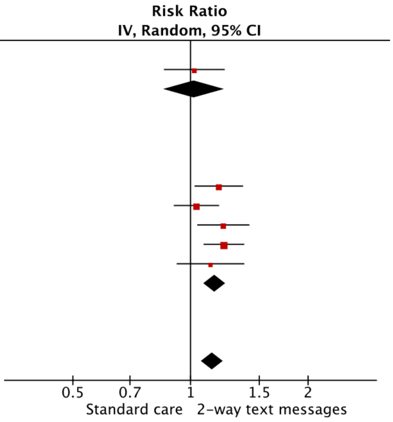

Figure D: Subgroup analysis: Low risk of bias versus high risk of bias on medicine adherence

Study or Subgroup	log[Risk Ratio]	SE	2-way text messages Total	Standard care Total	Weight	Risk Ratio IV, Random, 95% CI
**5.7.1 Hypertension**						
Bobrow 2016	−0.0153	0.0297	458	457	38.1%	0.98 [0.93, 1.04]
**Subtotal (95% CI)**			**458**	**457**	**38.1%**	**0.98 [0.93, 1.04]**
Heterogeneity: Not applicable						
Test for overall effect: Z = 0.52 (P = 0.61)						
**5.7.2 HIV**						
Leiby 2016	0.193	0.1696	533	550	5.5%	1.21 [0.87, 1.69]
Odeny 2014	0.5098	0.2475	194	187	2.7%	1.66 [1.03, 2.70]
Odeny 2019	0.1133	0.0734	548	747	19.4%	1.12 [0.97, 1.29]
Van der Kop 2018	−0.0228	0.0375	349	351	34.3%	0.98 [0.91, 1.05]
**Subtotal (95% CI)**			**1624**	**1835**	**61.9%**	**1.10 [0.95, 1.29]**
Heterogeneity: $Tau^2 = 0.01$; $Chi^2 = 7.87$, df = 3 (P = 0.05); $I^2 = 62\%$						
Test for overall effect: Z = 1.27 (P = 0.21)						
**Total (95% CI)**			**2082**	**2292**	**100.0%**	**1.03 [0.95, 1.12]**
Heterogeneity: $Tau^2 = 0.00$; $Chi^2 = 8.55$, df = 4 (P = 0.07); $I^2 = 53\%$						
Test for overall effect: Z = 0.78 (P = 0.44)						
Test for subgroup differences: $Chi^2 = 1.87$, df = 1 (P = 0.17), $I^2 = 46.4\%$						

Risk Ratio
IV, Random, 95% CI

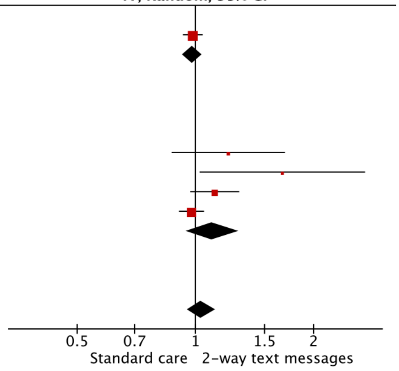

Figure E: Subgroup analysis: Comparison of clinical areas on appointment attendance

Study or Subgroup	log[Risk Ratio]	SE	2-way text messages Total	Standard care Total	Weight	Risk Ratio IV, Random, 95% CI
**5.15.1 Hypertension**						
Bobrow 2016	0.1669	0.073	458	457	17.9%	1.18 [1.02, 1.36]
**Subtotal (95% CI)**			**458**	**457**	**17.9%**	**1.18 [1.02, 1.36]**
Heterogeneity: Not applicable						
Test for overall effect: Z = 2.29 (P = 0.02)						
**5.15.2 HIV**						
Kassaye 2016	0.0351	0.0682	280	270	20.3%	1.04 [0.91, 1.18]
Lester 2010	0.1933	0.0785	273	265	15.7%	1.21 [1.04, 1.42]
Mbuagbaw 2012	0.0226	0.0915	101	99	11.8%	1.02 [0.85, 1.22]
Sumari-De Boer 2021	0.1178	0.1017	80	81	9.7%	1.13 [0.92, 1.37]
**Subtotal (95% CI)**			**734**	**715**	**57.4%**	**1.09 [1.01, 1.19]**
Heterogeneity: $Tau^2 = 0.00$; $Chi^2 = 3.00$, df = 3 (P = 0.39); $I^2 = 0\%$						
Test for overall effect: Z = 2.18 (P = 0.03)						
**5.15.3 Malaria**						
Modrek 2014	0.1971	0.0612	213	206	24.6%	1.22 [1.08, 1.37]
**Subtotal (95% CI)**			**213**	**206**	**24.6%**	**1.22 [1.08, 1.37]**
Heterogeneity: Not applicable						
Test for overall effect: Z = 3.22 (P = 0.001)						
**Total (95% CI)**			**1405**	**1378**	**100.0%**	**1.14 [1.07, 1.21]**
Heterogeneity: $Tau^2 = 0.00$; $Chi^2 = 5.44$, df = 5 (P = 0.37); $I^2 = 8\%$						
Test for overall effect: Z = 4.02 (P < 0.0001)						
Test for subgroup differences: $Chi^2 = 2.44$, df = 2 (P = 0.30), $I^2 = 18.0\%$						

Risk Ratio
IV, Random, 95% CI

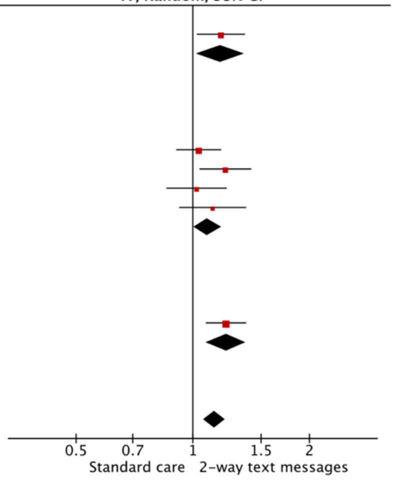

Figure F: Subgroup analysis: Comparison of clinical areas on medicine adherence

Study or Subgroup	log[Risk Ratio]	SE	2-way text messages Total	Standard care Total	Weight	Risk Ratio IV, Random, 95% CI
**5.3.2 No HCP contact**						
Bobrow 2016	−0.0153	0.0297	458	457	38.1%	0.98 [0.93, 1.04]
Leiby 2016	0.193	0.1696	533	550	5.5%	1.21 [0.87, 1.69]
Van der Kop 2018	−0.0228	0.0375	349	351	34.3%	0.98 [0.91, 1.05]
**Subtotal (95% CI)**			**1340**	**1358**	**77.9%**	**0.99 [0.94, 1.03]**
Heterogeneity: $Tau^2 = 0.00$; $Chi^2 = 1.55$, df = 2 (P = 0.46); $I^2 = 0\%$						
Test for overall effect: Z = 0.62 (P = 0.54)						
**5.3.3 HCP contact**						
Odeny 2014	0.5098	0.2475	194	187	2.7%	1.66 [1.03, 2.70]
Odeny 2019	0.1133	0.0734	548	747	19.4%	1.12 [0.97, 1.29]
**Subtotal (95% CI)**			**742**	**934**	**22.1%**	**1.27 [0.89, 1.83]**
Heterogeneity: $Tau^2 = 0.05$; $Chi^2 = 2.36$, df = 1 (P = 0.12); $I^2 = 58\%$						
Test for overall effect: Z = 1.30 (P = 0.19)						
**Total (95% CI)**			**2082**	**2292**	**100.0%**	**1.03 [0.95, 1.12]**
Heterogeneity: $Tau^2 = 0.00$; $Chi^2 = 8.55$, df = 4 (P = 0.07); $I^2 = 53\%$						
Test for overall effect: Z = 0.78 (P = 0.44)						
Test for subgroup differences: $Chi^2 = 1.87$, df = 1 (P = 0.17), $I^2 = 46.5\%$						

Risk Ratio
IV, Random, 95% CI

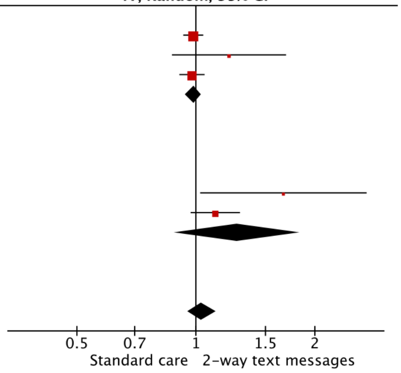

Figure G: Subgroup analysis: Comparison of interaction with HCP through a phone call or text message only on appointment attendance

Study or Subgroup	log[Risk Ratio]	SE	2-way text messages Total	Standard care Total	Weight	Risk Ratio IV, Random, 95% CI
**5.16.1 No HCP contact**						
Bobrow 2016	0.1669	0.073	458	457	17.9%	1.18 [1.02, 1.36]
Lester 2010	0.1933	0.0785	273	265	15.7%	1.21 [1.04, 1.42]
Modrek 2014	0.1971	0.0612	213	206	24.6%	1.22 [1.08, 1.37]
Sumari-De Boer 2021	0.1178	0.1017	80	81	9.7%	1.13 [0.92, 1.37]
**Subtotal (95% CI)**			**1024**	**1009**	**67.9%**	**1.19 [1.11, 1.29]**
Heterogeneity: Tau² = 0.00; Chi² = 0.51, df = 3 (P = 0.92); I² = 0%						
Test for overall effect: Z = 4.74 (P < 0.00001)						
**5.16.2 HCP contact**						
Kassaye 2016	0.0351	0.0682	280	270	20.3%	1.04 [0.91, 1.18]
Mbuagbaw 2012	0.0226	0.0915	101	99	11.8%	1.02 [0.85, 1.22]
**Subtotal (95% CI)**			**381**	**369**	**32.1%**	**1.03 [0.93, 1.15]**
Heterogeneity: Tau² = 0.00; Chi² = 0.01, df = 1 (P = 0.91); I² = 0%						
Test for overall effect: Z = 0.56 (P = 0.58)						
**Total (95% CI)**			**1405**	**1378**	**100.0%**	**1.14 [1.07, 1.21]**

Heterogeneity: Tau² = 0.00; Chi² = 5.44, df = 5 (P = 0.37); I² = 8%
Test for overall effect: Z = 4.02 (P < 0.0001)
Test for subgroup differences: Chi² = 4.92, df = 1 (P = 0.03), I² = 79.7%

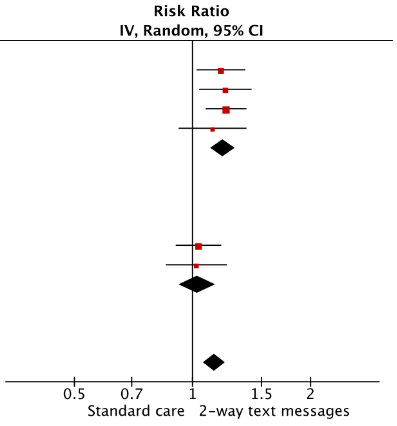

Figure H: Subgroup analysis: Comparison of interaction with HCP through a phone call or text message only on medicine adherence

Study or Subgroup	log[Risk Ratio]	SE	2-way text messages Total	Standard care Total	Weight	Risk Ratio IV, Random, 95% CI
Bobrow 2016	−0.0153	0.0297	458	457	22.1%	0.98 [0.93, 1.04]
Leiby 2016	0.193	0.1696	533	550	10.4%	1.21 [0.87, 1.69]
Lund 2014	0.3429	0.0526	1311	1239	20.6%	1.41 [1.27, 1.56]
Odeny 2014	0.5098	0.2475	194	187	6.5%	1.66 [1.03, 2.70]
Odeny 2019	0.1133	0.0734	548	747	18.7%	1.12 [0.97, 1.29]
Van der Kop 2018	−0.0228	0.0375	349	351	21.7%	0.98 [0.91, 1.05]
**Total (95% CI)**			**3393**	**3531**	**100.0%**	**1.15 [0.99, 1.33]**

Heterogeneity: Tau² = 0.02; Chi² = 44.51, df = 5 (P < 0.00001); I² = 89%
Test for overall effect: Z = 1.84 (P = 0.07)

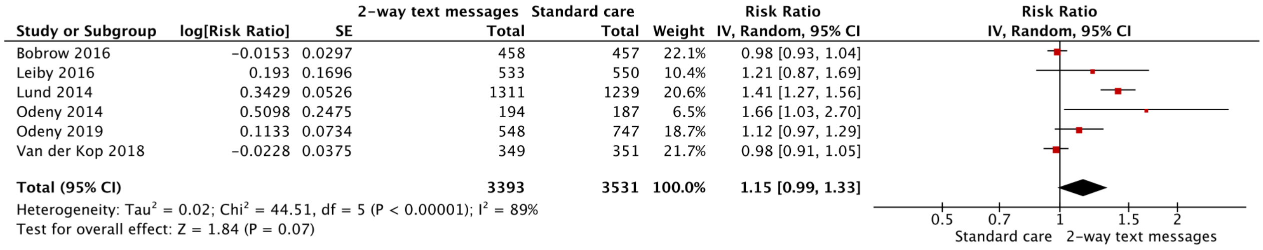

Figure I: Sensitivity analysis: Pooled RR on appointment attendance including Lund 2014

Study or Subgroup	log[Risk Ratio]	SE	2-way text messages Total	Standard care Total	Weight	Risk Ratio IV, Random, 95% CI
Bobrow 2016	−0.0153	0.0297	458	457	48.2%	0.98 [0.93, 1.04]
Leiby 2016	0.193	0.1696	533	550	6.1%	1.21 [0.87, 1.69]
Odeny 2014	0.5098	0.2475	194	187	3.0%	1.66 [1.03, 2.70]
Van der Kop 2018	−0.0228	0.0375	349	351	42.7%	0.98 [0.91, 1.05]
**Total (95% CI)**			**1534**	**1545**	**100.0%**	**1.01 [0.93, 1.10]**

Heterogeneity: Tau² = 0.00; Chi² = 5.99, df = 3 (P = 0.11); I² = 50%
Test for overall effect: Z = 0.23 (P = 0.82)

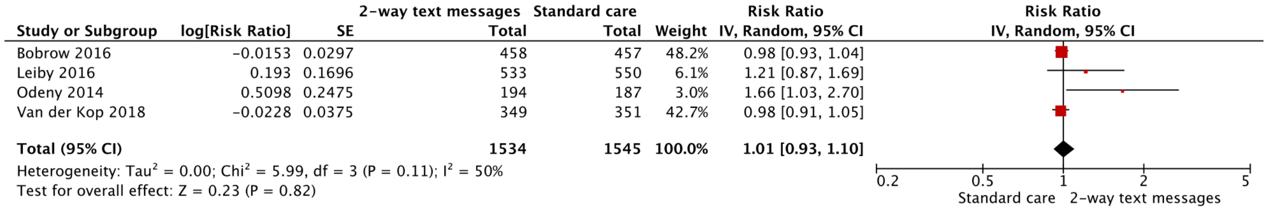

Figure J: Sensitivity analysis: Cluster randomized trials removed. Two-way SMS versus standard care on appointment attendance.

Study or Subgroup	log[Risk Ratio]	SE	2-way text messages Total	Standard care Total	Weight	Risk Ratio IV, Random, 95% CI
Bobrow 2016	0.1669	0.073	458	457	22.5%	1.18 [1.02, 1.36]
Lester 2010	0.1933	0.0785	273	265	19.5%	1.21 [1.04, 1.42]
Mbuagbaw 2012	0.0226	0.0915	101	99	14.3%	1.02 [0.85, 1.22]
Modrek 2014	0.1971	0.0612	213	206	32.0%	1.22 [1.08, 1.37]
Sumari-De Boer 2021	0.1178	0.1017	80	81	11.6%	1.13 [0.92, 1.37]
**Total (95% CI)**			**1125**	**1108**	**100.0%**	**1.17 [1.09, 1.25]**

Heterogeneity: Tau² = 0.00; Chi² = 2.97, df = 4 (P = 0.56); I² = 0%
Test for overall effect: Z = 4.48 (P < 0.00001)

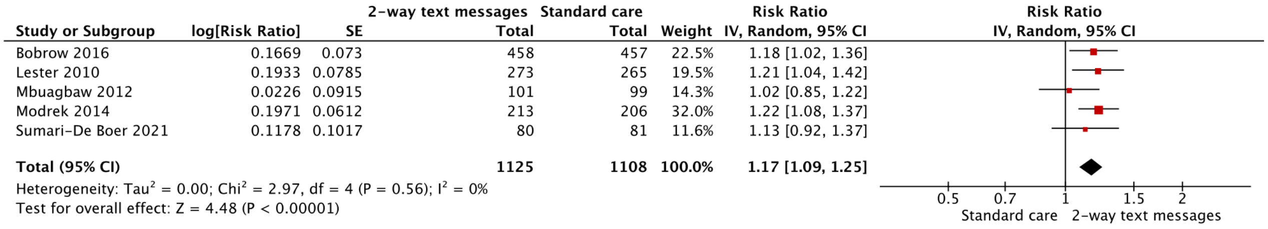

Figure K: Sensitivity analysis: Cluster randomized trials removed. Two-way SMS versus standard care on medicine adherence.

Study or Subgroup	log[Risk Ratio]	SE	2-way SMS Total	Standard care Total	Weight	Risk Ratio IV, Fixed, 95% CI
Van der Kop 2018	−0.0228	0.0375	349	351	34.3%	0.98 [0.91, 1.05]
Bobrow 2016	−0.0153	0.0297	458	457	54.6%	0.98 [0.93, 1.04]
Odeny 2019	0.116	0.0747	596	747	8.6%	1.12 [0.97, 1.30]
Leiby 2016	0.193	0.1696	533	550	1.7%	1.21 [0.87, 1.69]
Odeny 2014	0.5098	0.2475	194	187	0.8%	1.66 [1.03, 2.70]
**Total (95% CI)**			**2130**	**2292**	**100.0%**	**1.00 [0.96, 1.05]**

Heterogeneity: $Chi^2 = 8.58$, df = 4 (P = 0.07); $I^2 = 53\%$
Test for overall effect: Z = 0.05 (P = 0.96)

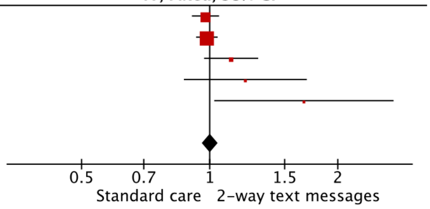

Figure L: Sensitivity analysis: Fixed effect model of two-way SMS versus standard care on appointment attendance.

Study or Subgroup	log[Risk Ratio]	SE	2-way text messages Total	Standard care Total	Weight	Risk Ratio IV, Fixed, 95% CI
Bobrow 2016	0.1669	0.073	458	457	17.9%	1.18 [1.02, 1.36]
Kassaye 2016	0.0351	0.0682	280	270	20.5%	1.04 [0.91, 1.18]
Lester 2010	0.1933	0.0785	273	265	15.5%	1.21 [1.04, 1.42]
Mbuagbaw 2012	0.0226	0.0915	101	99	11.4%	1.02 [0.85, 1.22]
Modrek 2014	0.1971	0.0612	213	206	25.5%	1.22 [1.08, 1.37]
Sumari-De Boer 2021	0.1178	0.1017	80	81	9.2%	1.13 [0.92, 1.37]
**Total (95% CI)**			**1405**	**1378**	**100.0%**	**1.14 [1.07, 1.21]**

Heterogeneity: $Chi^2 = 5.44$, df = 5 (P = 0.37); $I^2 = 8\%$
Test for overall effect: Z = 4.23 (P < 0.0001)

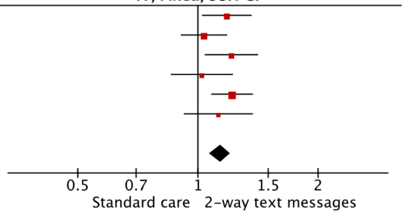

Figure K: Sensitivity analysis: Fixed effect model of two-way SMS versus standard care on medicine adherence
