# Supplementary material for: Two-way text message interventions and healthcare outcomes in Africa: Systematic review of randomized trials with meta-analyses on appointment attendance and medicine adherence
Source: PLoS One. 2022 Apr 14;17(4):e0266717. doi: 10.1371/journal.pone.0266717 (PMC9009629; doi:10.1371/journal.pone.0266717)
Supplement: S5 File — (PDF) [file pone.0266717.s005.pdf]

S3 File: Risk of bias assessment

Abiodun, 2020

**Bias**<br>***Outcome: Adherence***	**Authors' judgement**	**Support for judgement**
**Random sequence generation (selection bias)**	Low risk	"Random allocation was achieved through a computer-generated simple randomization scheme.»
**Allocation concealment (selection bias)**	Low risk	«the participants were randomly allocated to intervention and control groups by the database administrator.»
**Blinding of participants and personnel (performance bias)**	Low risk	Single-blind. "The investigators, data manager, research assistants, counselors, and other project staff were blinded throughout the study.»
**Blinding of outcome assessment (detection bias)**	Low risk	"The independentmedical internet technology firm was responsible for randomization of participants and the management of sending SMS to the participants and collecting responses from them. After the scientific review had been completed, at the point of analysis, a list of participants' unique identifiers in two groups was sent to the data analysts without specifyingwhichwas the investigation group and which was the control group."
**Incomplete outcome data (attrition bias)**	Low risk	"The study lost three participants to follow-up (two after the first visit, one after the third visit) while 209 adolescents completed the research. There was no statistically significant difference in the major demographic characteristics (age, gender, and education) between those who completed the study and those who did not."
**Selective reporting (reporting bias)**	Low risk	Protocol registered at clinicaltrials.gov in 2018 (NCT03394391). All specified outcomes in protocol reported in article.

Bobrow 2016

**Bias**<br>*Outcome: Adherence*	**Authors' judgement**	**Support for judgement**
**Random sequence generation (selection bias)**	Low risk	"A software algorithm assigned participants independently of the research team to information-only adherence support, interactive adherence support, or usual care in a 1:1:1 ratio using a nondeterministic minimization algorithm to ensure balance between groups with respect to age, sex, baseline SBP, years with hypertension, and recent clinic attendance"
**Allocation concealment (selection bias)**	Unclear	"All trial staff were masked to treatment allocation"
**Blinding of participants and personnel (performance bias)**	Low risk	"Researchers and clinicians were not aware of randomization assignment, were trained not to ask patients about the content of messages, and were unable to determine randomization group from casual comments by participants"
**Blinding of outcome assessment (detection bias)**	Low risk	"Blood pressure at 12 months from baseline measured with a validated oscillometric device. Blood pressure measurements were automated, and data were captured directly to the trial database. Trial statisticians, researchers, clinic staff, and research assistants who collected outcome data were masked to allocated interventions until the trial database was locked"
**Incomplete outcome data (attrition bias)**	Low risk	"92% outcome data. Attrition rates did not differ significantly between groups. All analyses were performed on an intention-to-treat basis, and outcomes were analysed using a mixed effect model. The model was adjusted for baseline systolic blood pressure and minimization factors. "

Kassaye, 2016

**Bias**<br>***Outcome: Adherence***	**Authors' judgement**	**Support for judgement**

**Random sequence generation (selection bias)**	Unclear risk	"Cluster randomization stratified by high volume and medium and low volumes". "A volume-stratified sampling method was applied to ensure inclusion of a representative sample of types of health facilities among the remaining facilities. All health facilities were randomly allocated to be an intervention or control site, stratified by high volume (hospitals) and medium and low volumes (health centers and dispensaries)." Method of randomization not described.
**Allocation concealment (selection bias)**	Unclear risk	Not described.
**Blinding of participants and personnel (performance bias)**	Unclear risk	Not described.
**Blinding of outcome assessment (detection bias)**	High risk	"Women underwent structured interviews at four visits to record self-reported adherence to antiretrovirals in the past week, number and mode of communication between the participant and health workers, time and place of delivery, infant feeding practices, and any intervening clinical outcomes." Was not blinded.
**Incomplete outcome data (attrition bias)**	High risk	"Estimation adjusted effects by logistic regression. Loss to follow up >10%" Higher proportion of people loss to follow-up in control group than intervention group (5 % more)
**Selective reporting (reporting bias)**	Low risk	"The study has been registered on ClinicalTrials.gov under the identifier NCT01645865." Data on all outcomes specified in the protocol are reported in article.

Leiby, 2016

**Bias** *Outcome: Attendance*	**Authors' judgement**	**Support for judgement**
**Random sequence generation (selection bias)**	Unclear risk	"Patients were randomly assigned to one of the 3 study arms". "The study sample was stratified by district (Lusaka or Chongwe), age (<18 or >18), and self-reported VMMC intention (within 2 months or not)." Method of randomisation not described.
**Allocation concealment (selection bias)**	Unclear risk	Not described
**Blinding of participants and personnel (performance bias)**	Unclear risk	"Participants were anonymous (numbers in platform). Subscriber phone numbers are not accessible to the counsellors or program managers, making the platform strictly confidential" No additional information about blinding of participants or personnel.
**Blinding of outcome assessment (detection bias)**	High risk	"The main method for collecting information from participants was SMS surveys + field team collected limited data (procedure date, partial phone numbers, age, and neighbourhood) from client intake forms."
**Selective reporting (reporting bias))**	High risk	Protocol submitted to the Registry for International Development Impact Evaluations in 2014. Not all specified secondary outcomes in protocol are listed in the article.

Lester, 2010

**Bias** *Outcome: Adherence*	**Authors' judgement**	**Support for judgement**
**Random sequence generation (selection bias)**	Low risk	"Individually randomised, parallel multisite controlled trial. Simple randomisation (1:1). A project statistician generated the randomisation numbers with a random number generating program."
**Allocation concealment (selection bias)**	Low risk	"Written allocation of assignment was sealed in individual opaque envelopes marked with study identification numbers, which were distributed to all three study clinics."
**Blinding of participants and personnel (performance bias)**	Low risk	"Randomisation, laboratory assays, and analyses were done by investigators masked to treatment allocation, study participants and clinic staff were not masked to treatment."
**Blinding of outcome assessment (detection bias)**	Low risk	"Women underwent structured interviews at four visits to record self-reported adherence to

		antiretrovirals in the past week, number and mode of communication between the participant and health workers, time and place of delivery, infant feeding practices, and any intervening clinical outcomes." Was not blinded.
**Incomplete outcome data (attrition bias)**	Low risk	«The analysis of primary outcomes was by intention to treat. The primary analyses were not adjusted, as prespecified and recommended. We also did a per-protocol (complete-case) analysis of the primary outcomes, in which only participants who had complete primary outcome data (self-reported adherence at 6 and 12 months and viral load at 12 months) were included. Heterogeneity of the effect of the intervention across subgroups was assessed by comparing logistic regression models with and without interaction term between treatment allocation and subgroup-defining variables» Loss to follow up is <95% in both intervention and control groups.
**Selective reporting (reporting bias)**	High risk	Protocol retrospectively registered in Clingov: NCT00830622, Registered on: January 28, 2009. Study start date: May 2007. Actual completion: March 2010.<br><br>Outcomes specified in protocol:<br>Primary outcome:<br>1.Adherence to ART and HIV RNA suppression.<br>Secondary outcomes:<br>1. Retention,<br>2. Quality of Life (SF-12)<br>3. Health (CD4, weight, progression to AIDS, all cause mortality)<br><br>Outcomes with reported results in article:<br>Primary outcome and all-cause mortality. Reported in article that all other pre-specified outcomes will be reported separately.

Lund, 2014

**Bias**<br>***Outcome: Adherence***	**Authors' judgement**	**Support for judgement**
**Random sequence generation (selection bias)**	Unclear risk	"Pragmatic cluster-randomised controlled trial. Primary healthcare facilities were assigned by simple random allocation to the mobile phone intervention. Stratified by district."<br>Method of randomisation not described.
**Allocation concealment (selection bias)**	Unclear risk	Not described.
**Blinding of participants and personnel (performance bias)**	High risk	"Neither study participants nor clinic staff were masked because of the nature of the intervention"
**Blinding of outcome assessment (detection bias)**	High risk	"The selected primary health care facility staff also functioned as research assistants. Clinical staff was not blinded"
**Selective reporting (reporting bias)**	High risk	Protocol retrospectively registered in Clingov: NCT01821222 published 2013, study start 2009. All specified outcomes in protocol, reported in article.

Mbuagbaw, 2012

**Bias**<br>***Outcome: Adherence***	**Authors' judgement**	**Support for judgement**
**Random sequence generation (selection bias)**	Low risk	"A computer-generated randomization list was established using random block sizes of 2, 4 and 6, by the Father Sean O'Sullivan Research Centre Biostatistics Unit at St Joseph's Healthcare/McMaster University (http://www.thecem.net/sjhsrn.php) in Canada."
**Allocation concealment (selection bias)**	Low risk	"The allocation codes were then sequentially affixed to the phone numbers of consecutively recruited participants by trained research staff at the YCH ATC. This sequence was sent to the research centre by email, and concealed in a password-

		protected computer until interventions were assigned”
**Blinding of participants and personnel (performance bias)**	Low risk	”From the point of enrolment, patients were identified only by their phone numbers and their sequential trial numbers. The interviewers transmitted the phone numbers of the enrolees to the research staff. The research staff responsible for allocation had access to the allocation codes and the phone numbers of participants. The program secretary responsible for sending the text messages received the allocations (SMS or No SMS) and corresponding phone numbers weekly. Only the participants were aware of their allocation.”
**Blinding of outcome assessment (detection bias)**	Low risk	“Adherence measured with VAS. Trained interviewers – blinded to group allocation – collected data using a pre-tested data collection form containing sociodemographic data, clinical information and adherence rates at baseline, 3 and 6 months” Single blinded, data analyst blinded.
**Incomplete outcome data (attrition bias)**	Low risk	“Intention-to-treat analysis. We also used multiple imputation techniques to handle missing data. Variables for which there was too much missing data to perform imputation were excluded from the analysis but are reported (CD4-T-lymphocyte cell count and viral load). All outcome variables had some degree of missing data ranging from 0 to 35%. Multiple imputation was used to create a new data set which was the average of five data sets of imputed values. This final data set was used for all analyses.” No difference in attrition in intervention and control group.
**Selective reporting (reporting bias)**	Low risk	All ways of measuring outcome were reported. All specified outcomes in protocol reported in article.<br>1. Pan-African Clinical Trials Registry; PACTR201011000261458. Nov 2010<br>2. Clinicaltrials.gov; NCT01247181. Nov 2010

Modrek, 2014

**Bias**<br>***Outcome: Adherence***	**Authors’ judgement**	**Support for judgement**
**Random sequence generation (selection bias)**	Unclear risk	“The protocol for treatment assignment entailed assigning every other survey to the SMS treatment group on the day of the baseline survey. To ensure that none of the survey staff would know who were chosen to receive the SMS, the study manager, who did not have any interaction with participants, randomly assigned surveys into the treatment group after the surveys were returned to the study office each day.” “Privately owned pharmacies and proprietary and patent medicine vendors (PPMVs) were initially randomly selected from the numerated sites within four local government areas and enrolled into the study.”<br>The method of randomisation is not described.
**Allocation concealment (selection bias)**	Unclear risk	Not describes
**Blinding of participants and personnel (performance bias)**	Low risk	«To ensure that none of the survey staff would know who were chosen to receive the SMS, the study manager, who did not have any interaction with participants, randomly assigned surveys into the treatment group after the surveys were returned to the study office each day.»
**Blinding of outcome assessment (detection bias)**	Low risk	Self-reported outcome. Survey staff blinded.
**Incomplete outcome data (attrition bias)**	Low risk	“Primary analysis estimates the intention-to-treat effect. Adjusted for unbalance across groups. Unbalanced variables are controlled for in subsequent regression model specifications. Of 465 adults enrolled adults, all of whom completed the baseline survey; 32 participants were not reached for follow-up. An additional eight surveys had duplicated survey numbers, so these observations were dropped, as it was impossible to tell which entry was correct. With these exclusions, 425

		participants remain who were reached in the follow-up phone survey. Only 419 observations are analysed. These “off-protocol” treatment assignments are taken into account in the statistical analyses of the data.” Outcome data for 91% of the participants.
**Selective reporting (reporting bias)**	Unclear risk	No protocol. Did not receive protocol on request. Analysis intentions are not available.

Odeny, 2019

**Bias** *Outcome: Adherence*	**Authors' judgement**	**Support for judgement**
**Baseline imbalance (recruitment bias)**	Low risk	“136 government health facilities, spread across 3 counties (Kisumu, Migori, and Homa Bay) in the Nyanza region of Kenya.[…] The top 20 clusters by patient volume (number of newly infected HIV-positive pregnant women in the prior 6 months) were selected for study inclusion.” “Stratification of study clusters based on volume and prior experience with implementing the intervention. Within each stratum, half of clinics were randomly assigned to begin implementing the intervention immediately, while the other half began implementing approximately 6 months later.”
**Random sequence generation (selection bias)**	Low risk	“An independent biostatistician at the University of Washington’s Center for AIDS Research generated the randomization sequence and assigned clusters to intervention start periods. Randomization was stratified by clinic volume and experience level.”
**Allocation concealment (selection bias)**	Unclear risk	“ In a pragmatic, cluster-randomized, stepped-wedge trial with 2 time periods of observation, we randomly allocated 10 clinics to begin implementing the intervention immediately and 10 clinics to begin implementing 6 months later.” “An independent biostatistician (…) assigned clusters to intervention start periods”. Concealment not described.
**Blinding of participants and personnel (performance bias)**	High risk	“Due to the nature of the intervention and the need to inform facilities of their participation, it was not possible to blind clusters, healthcare providers, investigators, data analysts, or individual participants to group assignments”
**Blinding of outcome assessment (detection bias)**	High risk	“Due to the nature of the intervention and the need to inform facilities of their participation, it was not possible to blind clusters, healthcare providers, investigators, data analysts, or individual participants to group assignments”
**Selective reporting (reporting bias)**	Low risk	Protocol registered at clinicaltrials.gov in 2015. (NCT02350140). All specified outcomes in protocol reported in article.

Odeny, 2014

**Bias** *Outcome: Adherence*	**Authors' judgement**	**Support for judgement**
**Random sequence generation (selection bias)**	Low risk	“Randomized to the intervention in a 1:1 Ratio, stratified by clinic. A block randomization scheme with variable block sizes was used”. No description of recruitment and baseline imbalance.
**Allocation concealment (selection bias)**	Low risk	“Intervention groups were assigned using sealed, opaque envelopes. Investigators and study staff were unaware of block numbers, sizes, or sequences.”
**Blinding of participants and personnel (performance bias)**	Low risk	“Investigators and study staff were unaware of block numbers, sizes, or sequences. Study staff called participants in the SMS arm weekly beginning at 38 weeks gestation to ascertain whether delivery had occurred. Delivery dates for participants in the control arm were abstracted from clinic records. If control women did not return, they were contacted either in person or by phone”

**Blinding of outcome assessment (detection bias)**	Unclear risk	"Postpartum retention in PMTCT was defined as return for at least one visit at the PMTCT or postnatal clinic within 8 weeks after delivery. Infant HIV testing was defined as obtaining a dried blood spot (DBS) sample for virological HIV testing within 8 weeks after birth." No further description of outcome assessment provided.
**Selective reporting (reporting bias)**	Unclear risk	No protocol. Did not receive protocol on request. Analysis intentions are not available.

Sumari-de Boer, 2021

**Bias** *Outcome: Adherence*	**Authors' judgement**	**Support for judgement**
**Random sequence generation (selection bias)**	Low risk	"Participants were subsequently randomized by using the randomization module in Redcap whereby the data manager assigned participants to theinterventions.»
**Allocation concealment (selection bias)**	High risk	"One month later, during the enrolment visit with the study nurse, viral load was measured, and participants allocated to the intervention arms were provided with anexplanation on how to use the DAT."
**Blinding of participants and personnel (performance bias)**	High risk	Not described clearely. "During the enrolment visits, participants were shown how to use the device." Due to the nature of the intervention, it seems that both participants and personnel were not blinded.
**Blinding of outcome assessment (detection bias)**	High risk	"Through a Web-based interface with authorized access, the study team could download adherence reports showing the number of SMS that had been sent, delivered, and replied to (SMS arm) or showing the pillbox openings (RTMM arm)." " Participants were asked about their opinion regarding their self-reported adherence since the previous visit (precontemplation), followed by showing an adherence report on which participants were asked to reflect (contemplation).»
**Incomplete outcome data (attrition bias)**	Low risk	"A modified intention-to-treat approach was used for primary analyses.31 We included only participants who came for a second visit after enrolment where outcome parameters on adherence data were collected the first time. We excluded patients who did not attend the second visit and for whom we were, thus, unable to collect the necessary data.»
**Selective reporting (reporting bias)**	Low risk	Protocol registered under PACTR201712002844286 in 2017, at cochranelibrary.com in 2019. Data on all outcomes specified in protocol reported in article.

Unger, 2018

**Bias** *Outcome: Adherence*	**Authors' judgement**	**Support for judgement**
**Random sequence generation (selection bias)**	Low risk	"Randomized using 1:1:1 allocation. An independent statistician generated a computer-generated randomisation list using random block sizes."
**Allocation concealment (selection bias)**	Low risk	"The allocation codes were placed in sequentially numbered, opaque, sealed envelopes and distributed by research staff. Envelopes were sequentially provided to participants at randomisation."
**Blinding of participants and personnel (performance bias)**	Low risk	"Randomisation allocation was unblinded to participants and study staff because the intervention required knowledge of group assignment"
**Blinding of outcome assessment (detection bias)**	Low risk	"Those obtaining and analysing follow-up data (DM, KR, JS and JU) were masked to group assignment."

**Incomplete outcome data (attrition bias)**	Low risk	«All analyses were intention-to-treat. For each of the primary outcomes of facility delivery, EBF, and contraception use, two sensitivity analyses were conducted to assess the influence of missing data. "Retention in the study to the 24-week visit was 86 (87%), 82 (83%), and 91 (91%) in the one-way, two-way, and control arms, respectively, and did not differ significantly by arm (P = 0.35 and P = 0.09 comparing the control arm with one-way and two-way arms, respectively)."
**Selective reporting (reporting bias)**	High risk	Protocol registered at clinicaltrials.gov in 2013 (NCT01894126). Did not report results for all secondary outcomes specified in protocol. Did not receive answer to our request for secondary outcome results.

Van der Kop, 2018

**Bias**<br>***Outcome: Adherence***	**Authors' judgement**	**Support for judgement**
**Random sequence generation (selection bias)**	Low risk	"Randomized in 1:1 ratio and random block sizes of 2, 4 and 6. Block sizes were not disclosed. An investigator was responsible for computerised sequence generation, and a research assistant for allocation concealment."
**Allocation concealment (selection bias)**	Low risk	"Allocations were sealed in individual, sequentially numbered opaque envelopes. After meeting inclusion criteria, consenting to participate, and completing baseline assessments, participants were assigned to a study group by the research nurse who opened one of the numbered envelopes to determine allocation."
**Blinding of participants and personnel (performance bias)**	Low risk	"The research nurses and participants were not masked to study group assignment because the intervention required overt participation". Clinic staff who collected data on primary and clinical outcomes were masked.
**Blinding of outcome assessment (detection bias)**	Low risk	"The data analyst and clinic staff (who collected data on primary and clinical outcomes), including lab technicians and community health workers who did the community tracing, were masked."
**Selective reporting (reporting bias)**	Low risk	Protocol registered at clinicaltrials.gov in 2012 (NCT01630304). Data on all outcomes specified in protocol and reported in article.
